# Three‐dimensional T1‐weighted gradient echo is a suitable alternative to two‐dimensional T1‐weighted spin echo for imaging the canine brain

**DOI:** 10.1111/vru.12774

**Published:** 2019-05-30

**Authors:** Kathryn L. Fleming, Thomas W. Maddox, Christopher M. R. Warren‐Smith

**Affiliations:** ^1^ School of Veterinary Science, Leahurst Campus University of Liverpool Neston UK; ^2^ School of Clinical Veterinary Science University of Bristol Bristol UK

**Keywords:** intracranial, isotropic, magnetic resonance imaging, MRI, volumetric

## Abstract

Volumetric imaging (VOL), a three‐dimensional magnetic resonance imaging (MRI) technique, has been described in the literature for evaluation of the human brain. It offers several advantages over conventional two‐dimensional (2D) spin echo (SE), allowing rapid, whole‐brain, isotropic imaging with submillimeter voxels. This retrospective, observational study compares the use of 2D T1‐weighted SE (T1W SE), with T1W VOL, for the evaluation of dogs with clinical signs of intracranial disease. Brain MRI images from 160 dogs who had T1W SE and T1W VOL sequences acquired pre‐ and postcontrast, were reviewed for presence and characteristics of intracranial lesions. Twenty‐nine of 160 patients were found to have intracranial lesions, all visible on both sequences. Significantly better grey‐white matter (GWM) differentiation was identified with T1W VOL (*P* < .001), with fair agreement between the two sequences (weighted *κ* = 0.35). Excluding a mild reduction in lesion intensity in three dogs precontrast on the T1W VOL images compared to T1W SE, and meningeal enhancement noted on the T1W VOL images in one dog, not identified on T1W SE, there was otherwise complete agreement between the two sequences. The T1W VOL sequence provided equivalent lesion evaluation and significantly improved GWM differentiation. Images acquired were of comparable diagnostic quality to those produced using a conventional T1W SE technique, for assessment of lesion appearance, number, location, mass effect, and postcontrast enhancement. T1W VOL, therefore, provides a suitable alternative T1W sequence for canine brain evaluation and can facilitate a reduction in total image acquisition time.

**Abbreviations**
2D: two‐dimensional
3D: three‐dimensional
CSF: cerebrospinal fluid
FLASH: fast low‐angle shot
GE: gradient echo
GWM: grey‐white matter
MP‐RAGE: magnetisation prepared rapid acquisition gradient echo
MPR: multiplanar reconstruction
MRI: magnetic resonance imaging
SE: spin echo
SNR: signal‐to‐noise ratio
TE: time to echo
TR: repetition time
T1W: T1‐weighted
T2W: T2‐weighted
VOL: volumetric imaging

## 1 INTRODUCTION

Magnetic resonance imaging is the modality of choice for imaging the canine brain, since it provides excellent tissue contrast resolution and anatomic detail in addition to multiplanar image acquisition, without the use of ionizing radiation.^1^, ^2^, ^3^ Increased caseloads have driven the need to acquire high‐quality images more rapidly. Shorter scan times facilitate a higher case throughput, but are also advantageous to the patient as the duration of anesthesia is reduced.

Traditionally, SE sequences have been used to acquire two‐dimensional (2D), cross‐sectional images of the canine brain. Gradient echo (GE) sequences typically use smaller flip angles than SE sequences (<90°) and a gradient to rephase the spins, rather than a 180° radiofrequency pulse. Use of a gradient increases the speed of rephasing, and smaller flip angles mean less time is required for relaxation; both strategies permit a shorter time to echo (TE) and repetition time (TR) than in SE, thus allowing studies to be performed more rapidly.^4^ Relatively short acquisition times permit the use of GE for three‐dimensional (3D) acquisition, also known as volumetric imaging (VOL). This is the simultaneous acquisition of data from an entire volume of tissue, in a single acquisition using a nonselective excitation pulse.^5^, ^6^, ^7^, ^8^ Use of GE to acquire VOL images of the brain has been frequently described in the human literature.^7^, ^9^, ^10^, ^11^, ^12^, ^13^

Spoiled GE sequences, for example, a fast low‐angle shot (FLASH) sequence, can be used to generate T1W VOL images, with good temporal and spatial resolution.^4^, ^6^, ^7^ Spoiled gradient echo uses a steady state, by using a very short TR and a medium flip angle, and a short TE to minimize T2* effects.^4^ T1W VOL images are acquired in very thin slices (<1 mm) ideally with isotropic voxels, without a slice gap, that allow image reformatting so that the voxels can be displayed as a new matrix of pixels without loss of spatial resolution.^5^, ^6^, ^14^ Therefore, only one volumetric acquisition is required, but images can be reformatted to allow assessment of transverse, sagittal, or dorsal planes,^5^, ^6^, ^15^ reducing total image acquisition time compared to acquiring 2D images in all three planes.^16^

In this study, we evaluate the use of a T1W VOL sequence compared to a conventional T1W SE sequence, for the routine assessment of canine brains. The appearance of lesions, enhancement, and grey‐white matter differentiation are appraised. The authors hypothesized that there would not be a significant difference identified between the two sequences and that T1W VOL imaging could be used in place of conventional T1W SE MRI, for routine canine brain imaging, providing equivalent lesion evaluation, while enabling a reduction in acquisition time.

## 2 MATERIALS AND METHODS

This was a retrospective, observational study. Ethical approval was granted by the Animal Welfare and Ethical Review Body of the University of Bristol. Dogs were selected from cases presented to the Small Animal Hospital, University of Bristol, between December 2015 and July 2016, by a radiology resident (K.L.F.). For inclusion, patients needed to have undergone MRI to further investigate clinical signs referable to the brain, with acquisition of T1W SE and T1W VOL sequences of the brain, pre‐ and postcontrast. Patients were excluded if there was an absence of one or more of the required sequences, if they previously had a brain MRI, if the source of clinical signs was found to be extracranial, or if no clinical diagnosis was recorded, in an attempt to minimize the potential for inaccurate classification of cases.

Patient data collected included the following: breed, age, sex, presenting complaint, and clinical diagnosis. As part of the inclusion criteria, all imaging was performed with a 1.5T MRI unit (Magnetom Symphony, Siemens, Erlangen, Germany). A single channel head coil was used as a receiver coil. All dogs were positioned in dorsal recumbency under general anesthesia. All studies included the following sequences: T1W SE images in transverse, T1W VOL images in dorsal, and postcontrast T1W SE images and T1W VOL sequences acquired in the same planes as precontrast. Additional sequences routinely acquired included a T2‐weighted (T2W) sagittal and transverse, T2W FLAIR, and T2*W images.

Transverse images (T1W VOL reformatted by multiplanar reconstruction (MPR)), of each sequence were reviewed by one, board‐certified veterinary radiologist (C.W.S.) using DICOM viewer freeware (Horos, v2.0.1; The Horos Project; http://www.horosproject.org). The reviewer was blinded to the history, patient signalment, and clinical or neurological examination findings. The cases were anonymized and viewed in a randomized order. The T1W SE images were reviewed separately to the T1W VOL images for each patient with the review of all of the T1W SE images performed over approximately 7 days and subsequently of the T1W VOL images over a similar time period. There was an interval of greater than 7 days between review of the T1W SE and the T1W VOL images. The image order was altered between the two readings (initially by case number order and then by acquisition date order). A standardized evaluation form was created for image analysis. Several parameters were identified (by C.W.S. and K.L.F.), which were considered important in the assessment of intracranial lesions that could aid presumptive diagnosis. The reviewer subjectively evaluated the images for: (a) lesion appearance precontrast (hyperintense, isointense, hypointense, or none seen); (b) number of lesions (single or multiple); (c) lesion location (intra‐axial or extra‐axial); (d) presence of mass effect (yes or no); (e) lesion enhancement postcontrast (homogeneous, heterogeneous, peripheral, or nonenhancing); (f) grey‐white matter (GWM) differentiation (minimal, moderate, marked), and (g) presence of meningeal enhancement (yes or no).

With respect to the GWM differentiation, minimal was defined as the following: no or very little difference in intensity between the grey and white matter, with the two virtually isointense and margins of the white matter hard to delineate. Moderate was defined as the following: a subtle but discernible difference in intensity evident between the grey and white matter, with white matter hyperintense to grey matter, but the margins of the white matter still not clearly defined at all times. Marked was defined as the following: a clear difference in intensity between the grey and white matter, with white matter obviously hyperintense to the grey matter and the margins of white matter well defined.

For the purposes of evaluation, the cases were categorized by the radiology resident (K.L.F.). Patients were grouped by their presenting complaint: (a) ataxia (ataxia/circling/head tilt/gait alteration/loss of balance); (b) seizures (or partial seizures); (c) weakness/collapse/tremors; (d) facial nerve paralysis; (e) blindness, or (f) other. The clinical diagnosis was recorded for each patient. Clinical diagnosis was defined as: that reached after appraisal of the history, neurological assessment, interpretation of all MRI sequences (not just T1W) and any relevant clinical data (bloods, cerebrospinal fluid (CSF) analysis, and where available histopathology), following the exclusion of other disease processes. The diagnoses were grouped as: (a) idiopathic; (b) neoplasia (eg, meningioma, glioma, choroid plexus tumor); (c) inflammatory/infectious (eg, non‐infectious inflammatory meningoencephalitis (ie, granulomatous meningoencephalitis or necrotizing encephalitis), or bacterial meningitis); (d) vascular (eg, suspected cerebrovascular event/infarct or focal hemorrhage); (e) congenital (eg, Chiari‐like malformation or suspected epidermoid cyst), or (f) degenerative/metabolic/toxic/nutritional.

### 2.1 Statistical analysis

All statistical analyses were performed using dedicated statistical software (SPSS 20.0, SPSS Inc, Chicago, IL, USA) by an author experienced in statistical analysis (T.W.M.). Independent variables were derived from information obtained from the signalment data, presenting complaints and clinical diagnosis. Descriptive statistics were generated for all variables, with the categorical data amalgamated into appropriate groups if required (due to small groups sizes) and expressed as frequencies. Normality of distribution for age was also assessed via the Kolmogorov‐Smirnov and Shapiro‐Wilk tests.

The primary outcome considered was the proportion of studies identified as having marked GWM differentiation on the T1W SE and T1W VOL sequences. A McNemar test for paired data was used to evaluate differences in proportions between these sequences. Weighted Kappa (*κ*_w_) was used to measure the degree of agreement for GWM differentiation, where there were three ordinal categories. Cohen's Kappa (*κ*) was used as a standard measure of agreement between the two sequences for the presence of meningeal enhancement. Strength of agreement was evaluated according to the following Kappa values: ≤0.2 poor, 0.21‐0.4 fair, 0.41‐0.6 moderate, 0.61‐0.8 good, and 0.81‐1.00 very good agreement. The percentage of cases considered to be normal compared to those seen to have visible lesions was also calculated. Results were considered significant if *P* < .05.

Sample size calculations indicated that to detect a minimum difference of 10% between the proportion of T1W SE versus T1W VOL studies graded as having marked GWM distinction, with a power of 80% and a two‐sided significance of 5% and allowing for correlation of approximately 0.6 between paired observations, a minimum of 140 animals were required.

## 3 RESULTS

A total of 175 dogs were initially identified. Of these, 15 were excluded based on the previously detailed exclusion criteria. Thus, 160 dogs were included in analyses for the current study. The study population had a median age of 5 years (range 4 months to 14 years 10 months, with an interquartile range of 2 years 10 months to 8 years 4 months). There were 62 neutered females, 56 neutered males, 24 entire males, and 18 entire females. Forty breeds were represented, the most common of which were: cross‐breeds (28), Labradors (13), Boxers (10), Cavalier King Charles Spaniels (8), Cocker Spaniels (8), Jack Russell Terriers (8), French Bulldogs (7), and West Highland White Terriers (7). The weights of the included patients ranged from 3.8 kg to 69 kg (median weight = 18 kg).

Imaging parameters were tailored to each patient. The T1W SE images were acquired in a transverse plane; TR was between 376‐771 ms and TE was 10 ms. Slice thickness was 2.5‐3 mm in most cases, but was typically reduced to 2 mm in patients <10 kg, with an interslice gap of ≤0.9 mm. The acquisition time was approximately 3 min 30 s (range 3‐4 min). For the T1W VOL images, a FLASH sequence with chemical fat suppression was used. Images were acquired in a dorsal plane with a TR of 20 ms, TE of 9.53 ms, and a flip angle of 25°. The isotropic voxel size was 0.7‐0.9 mm depending on the patient size. The acquisition time was approximately 6 min 20 s (range 6‐7 min). The postcontrast sequences were usually run within 1 min of an intravenous bolus of 0.1 mmol/kg gadobenate dimeglumine (MultiHance 0.5 M, Bracco Imaging spa, Milan, Italy); individual variations in exact timing of the postcontrast image acquisition occurred due to anesthesia staff occasionally performing patient checks or adjusting equipment settings. The imaging parameters were kept the same, with T1 VOL images acquired first, immediately followed by the T1W SE.

The details of the patients’ presenting complaint/clinical signs and the clinical diagnoses are summarized in Tables 1 and 2. A summary of the MRI findings based on separate evaluation of the T1W SE and T1W VOL images is shown in Table 3. The percentage agreement between the two sequences is also detailed.

**Table 1:** The patients’ main presenting complaint/clinical sign, as detailed in the clinical records

Main presenting complaint/clinical sign	Number of patients	Percentage (%)
Seizures/partial seizures	71	44.4
Ataxia/circling/head tilt/gait alteration/loss of balance	40	25.0
Other	29	18.1
Weakness/collapse/tremors	13	8.1
Blindness	5	3.1
Facial nerve paralysis	2	1.3
	**160**	**100.0**

**Table 2:** The clinical diagnosis for each patient, as detailed in the patient records

Clinical diagnosis classification	Number of patients	Percentage (%)
Idiopathic	66	41.3
Inflammatory/infectious	27	16.9
Other (including: movement disorder, endocrinopathy, open)	26	16.3
Neoplasia	23	14.4
Vascular	8	5.0
Congenital	5	3.1
Degenerative/metabolic/toxic/nutritional	5	3.1
Total	**160**	**100.0**

The clinical diagnosis was defined as: that reached after appraisal of the history, neurological assessment, interpretation of all MRI sequences (not just T1W) and any relevant clinical data (bloods, cerebrospinal fluid (CSF) analysis and where available histopathology), following the exclusion of other disease processes.

**Table 3:** Summarized findings for each sequence showing the number (n)* and percentage (%) of patients exhibiting each feature evaluated and the number and percentage of patients in which there was agreement between the T1W SE and T1W VOL sequences

		T1W SE		T1W VOL		Sequences in agreement	
		n	%	n	%	n	%
Normal brain		131/160	*81.9*	131/160	*81.9*	131/160	*100*
Abnormal brain		29/160	*18.1*	29/160	*18.1*	29/29	*100*
Lesion appearance (pre‐contrast)	Hyperintense	3/160	*1.9*	2/160	*1.3*	2/3	*66.7*
	Isointense	7/160	*4.4*	6/160	*3.7*	5/8	*62.5*
	Hypointense	19/160	*11.9*	21/160	*13.1*	19/21	*90.5*
	None seen	131/160	*81.9*	131/160	*81.9*	131/131	*100*
Number of lesions	Single	22/29	*75.9*	22/29	*75.9*	22/22	*100*
	Multiple	7/29	*24.1*	7/29	*24.1*	7/7	*100*
Lesion location	Intra‐axial	24/29	*82.8*	24/29	*82.8*	24/24	*100*
	Extra‐axial	5/29	*17*	5/29	*17.2*	5/5	*100*
Presence of mass effect	Yes	22/29	*75.9*	22/29	*75.9*	22/22	*100*
	No	7/29	*24.1*	7/29	*24.1*	7/7	*100*
Enhancement (post‐contrast)	Homogeneous	8/29	*27.6*	8/29	*27.6*	8/8	*100*
	Heterogeneous	11/29	*37.9*	11/29	*37.9*	11/11	*100*
	Peripheral	6/29	*20.7*	6/29	*20.7*	6/6	*100*
	Non‐enhancing	4/29	*13.8*	4/29	*13.8*	4/4	*100*
GWM differentiation	Marked	21/160	*13.1*	110/160	*68.7*	18/113	*15.9*
	Moderate	121/160	*75.6*	47/160	*29.4*	32/136	*23.5*
	Minimal	18/160	*11.3*	3/160	*1.9*	2/19	*10.5*
Meningeal enhancement	Yes	5/160	*3.1*	6/160	*3.7*	5/6	*83.3*
	No	155/160	*96.9*	154/160	*96.3*	154/155	*99.4*

^*^When considering each sequence: for features evaluated in all patients n = 160; for features that could only be evaluated when a lesion was present n = 29 (ie, representing the total number of patients where lesions were identified with each sequence).

**Figure 1:**
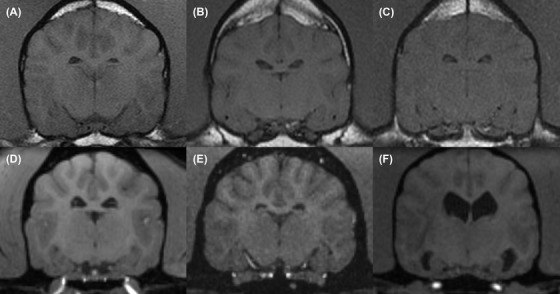
Transverse T1‐weighted SE (T1W SE) (A, B, and C) and T1‐weighted volumetric (T1W VOL) MRI (D, E, and F). Examples of patients subjectively classified as having either: marked (A and D), moderate (B and E), or minimal (C and F) grey‐white‐matter (GWM) differentiation

**Figure 2:**
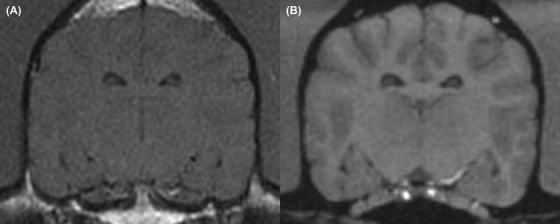
Transverse MRI of a case with disparities identified in grey‐white‐matter differentiation between the two sequences. A, T1‐weighted spin echo image with minimal grey‐white‐matter differentiation and B, T1‐weighted volumetric image with marked grey‐white‐matter differentiation. (A 1 year old, female entire, Staffordshire Bull Terrier, presented for seizures; MRI was unremarkable)

The most notable difference identified between the two sequences was the GWM differentiation. One hundred ten of 160 patients (68.7%) showed marked GWM differentiation on the T1W VOL images in contrast to only 21/160 (13.1%) classified as marked on the T1W SE images. The T1W VOL sequence enabled significantly better differentiation of the grey and white matter than the T1W SE sequence (*P* < .001). Agreement between the two sequences for GWM differentiation was fair (weighted *κ* = 0.35). Only three of 160 patients (1.9%) had minimal GWM differentiation on the T1W VOL sequences; whereas with the T1W SE sequence 18/160 (11.3%) were classified as minimal. With the T1W SE sequence, the majority of patients, 121/160 (75.6%) exhibited moderate GWM differentiation. Examples of different GWM differentiation are shown in Figure 1. An example of the difference seen in GWM differentiation between the two sequences is shown in Figure 2.

On both the T1W SE and T1W VOL sequences 29/160 patients (18.1%) had lesions identified. The remaining 131/160 (81.9%) had no lesion identified pre‐ or postcontrast with either sequence (ie, the brain was considered normal). With the T1W SE precontrast, three patients’ lesions were classified as hyperintense, seven as isointense, and 19 as hypointense. Assessment of the T1W VOL images precontrast identified two patients with lesions that were classified as hyperintense, six as isointense, and 21 as hypointense. There were two of three cases in agreement between the two sequences for the hyperintense classification, five of eight for isointense, and 19/21 in agreement classified as hypointense.

Twenty‐nine of 160 patients had lesions identified, all visible on both sequences. No differences were identified between the two sequences for determination of the number of lesions (single or multiple), the assessment of the lesion location, the presence of a mass effect, or the enhancement pattern. Both the T1W SE and T1W VOL identified 22/29 patients (75.9%) with single lesions and seven of 29 (24.1%) with multiple lesions. The lesion location was shown to be intra‐axial in 24/29 (82.8%) of the patients on both sequences and extra‐axial in five of 29 (17.2%) patients. A mass effect was identified in 22/29 dogs (75.9%) on both the T1W SE and T1W VOL sequences and was absent in seven of 29 (24.1%) dogs. Contrast enhancement was heterogeneous in 11/29 (37.9%), homogeneous in eight of 29 (27.6%), and peripheral in six of 29 (20.7%), while four of 29 (13.8%) of the patients’ lesions were noncontrast enhancing.

A high level of agreement for presence or absence of meningeal enhancement was demonstrated between the two sequences (*κ* = 0.906). There was no meningeal enhancement in 155/160 (96.9%) of patients on the T1 SE and 154/160 patients (96.3%) on T1W VOL sequence. Meningeal enhancement was present in both sequences in five of 160 (3.1%) and seen on T1W VOL imaging only in just one of 160 (0.06%) patients.

**Figure 3:**
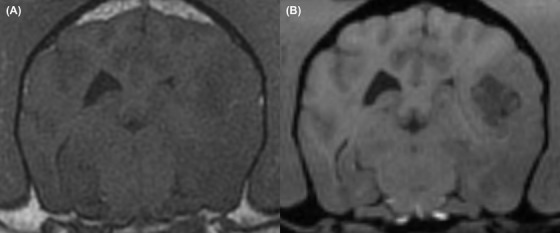
Transverse T1‐weighted spin echo (A) and T1‐weighted volumetric (B) MRI from a patient where there was a difference in the classification of the lesion intensity precontrast between the two sequences. A, On T1‐weighted spin echo the lesion was classified as isointense. B, On T1‐weighted volumetric images the lesion was classified as hypointense

**Figure 4:**
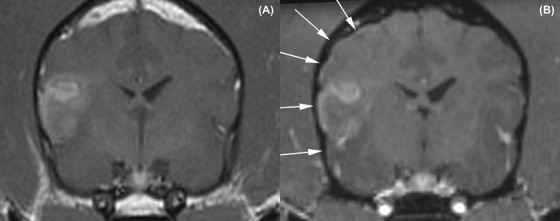
Transverse T1‐weighted spin echo (A) and T1‐weighted volumetric (B) MRI demonstrating a disparity regarding the classification of meningeal enhancement recorded in this patient. A, Absence of meningeal enhancement on T1‐weighted spin echo. B, Subtle pachymeningeal enhancement (indicated by arrows) associated with lesion, seen on T1‐weighted volumetric image

Discounting the GWM differentiation, only four of 160 patients showed a lack of agreement between the T1W SE and T1W VOL sequences. In three out of four of these patients, the precontrast relative signal intensity was lower on T1W VOL than T1W SE. In one patient, the lesion altered from hyperintense on T1W SE to isointense on T1W VOL (a 3‐year‐old male neutered Boxer, with a hemorrhagic infarct in the right olfactory lobe, diagnosed with *Angiostrongylus vasorum*). In two other patients, there was a change in classification from iso‐ to hypointense, when evaluated on the T1W VOL sequence instead of the T1W SE sequence (an 8‐year‐old female neutered Cavalier King Charles Spaniel, with a mass in the left cerebral hemisphere, most likely a neoplastic metastasis from previous mammary neoplasia (see Figure 3); also a 13‐year‐old female neutered Patterdale Terrier cross, with a hemorrhagic mass lesion within the right forebrain). The fourth patient was a 5‐year‐old female neutered Lurcher presented for blindness. She was found to have a single, intra‐axial, right parietal lobe hemorrhagic mass lesion, thought most likely to be neoplastic; it was hyperechoic precontrast, heterogeneously enhancing postcontrast, exerting a mass effect on both sequences. The inconsistency between the two sequences was the meningeal enhancement (of the pachymeninges) recorded as absent on the T1W SE sequence, yet present on the T1W VOL. (see Figure 4).

## 4 DISCUSSION

The findings of our study demonstrated that the T1W VOL sequence provided equivalent lesion evaluation to T1W SE imaging, for assessment of lesion appearance and enhancement. Additionally, significantly improved GWM differentiation was identified with the T1W VOL images compared to T1W SE. As hypothesized T1W VOL imaging can, therefore, provide a suitable alternative to conventional T1W SE MRI, for routine evaluation of the canine brain.

Volumetric imaging has several advantages over conventional 2D imaging.^5^, ^6^, ^7^, ^8^ The slice thickness can be much less than in conventional imaging, ≤0.9 mm in this study compared to 2‐3 mm for the SE sequence, permitting high spatial resolution. Thinner slices and the lack of slice gap can lessen the likelihood of very small lesions being missed, and reduce partial volume effects. In 2D imaging, where acquisition is performed one slice at a time, slice thickness will affect the signal‐to‐noise ratio (SNR). In VOL, data are acquired simultaneously from an entire volume of tissue in a single acquisition and divided into slices by a slice select gradient, in a process known as slice encoding.^4^ Since a whole volume of tissue is excited and there are no gaps, the SNR is increased.^4^ Additionally because the data are collected from a slab of tissue rather than a single slice, this can be reformatted using MPR to allow assessment of the region of interest in any plane.^5^, ^6^, ^10^ The use of small isotropic voxels (such as in this study) gives MPR's with high spatial resolution, which is equal regardless of plane.^4^, ^16^ This allows detailed evaluation of the brain and may permit improved detection of small intracranial lesions compared with 2D SE imaging.^17^, ^18^, ^19^ The use of VOL imaging to acquire 3D data sets, negates the need for supplemental sequences in orthoganol imaging planes, reducing acquisition time compared to acquisition of 2D images in all three planes.^5^, ^6^, ^7^, ^10^, ^16^

The T1W VOL sequence used in this study was a FLASH sequence, the use of which has been described in the human literature to obtain high resolution, very thin section, T1W images of the central nervous system.^6^ This sequence takes advantage of excitation pulses with small flip angles that speeds up acquisition time, by reducing the time between successive TR, without sacrificing spatial resolution or SNR.^5^, ^6^, ^20^ In contrast to SE, use of the low flip angle means much of the longitudinal magnetization remains unaffected and thus is available for immediate subsequent excitations.^20^ FLASH images can be acquired in 3D using a nonselective radiofrequency pulse and replacing the slice selection gradient with an additional phase‐encoding gradient, perpendicular to the other gradients, which separates the slices according to their phase value along the gradient.^4^, ^20^ With 3D imaging, data for the whole region of interest are collected throughout the image acquisition period.^6^

In this study, the T1W VOL sequence produced brain images with significantly better GWM differentiation than the images acquired using the T1W SE sequence (*P* < .001). Improved GWM differentiation permits more detailed evaluation of the internal structure of the brain and may aid the localization and characterization of lesions/pathology, especially those resulting in a reduction in normal GWM distinction. Evidence from the human literature corroborates our finding of a superior GWM differentiation using a T1W VOL technique.^6^, ^7^, ^9^, ^12^ The greater GWM differentiation results from stronger T1W contrast achieved by the GE sequence.^21^ These studies all employ a short‐TR GE technique using a magnetization‐preparation pulse (MP‐RAGE), a 3D‐Turbo FLASH technique, to produce T1W images.^21^ No apparent difference in image quality was demonstrated between images acquired using a 3D magnetisation prepared rapid acquisition gradient echo (MP‐RAGE) and 3D FLASH techniques in the study by Runge et al^22^ so the findings of these studies can be considered comparable to those in our study.

Not only did our study demonstrate that GWM differentiation was significantly better with the T1W VOL sequence, but also that lesion identification was equivalent. There was 100% agreement between the two sequences for identification of the presence of a lesion, that is, 29/160 patients were found to have a lesion(s) on T1W VOL and the same 29 had a lesion(s) identified on the T1W SE images. Additionally, there was complete (100%) agreement between the two sequences for classification of lesion number (single or multiple), lesion location (intra‐ or extra‐axial), presence or absence of mass effect, and the enhancement pattern postcontrast. This is consistent with the findings of human studies that have demonstrated that T1 VOL performs similarly to T1W SE for lesion detection in the brain.^7^, ^11^, ^13^ van den Hauwe et al^7^ reported that more lesions were identified with T1W VOL imaging in both patients with neoplastic and nonneoplastic disease, but no significant difference in lesion conspicuity between gadolinium‐enhanced MP‐RAGE and T1W SE images was found.

The degree of alteration in lesion precontrast signal intensity in the three patients is likely to be inconsequential and would not be expected to significantly alter the clinical diagnosis. It is postulated that in two patients the reduction in intensity may be a result of the altered appearance of hemorrhage on GE sequences compared to SE. In the patient where a difference was noted in the presence of meningeal enhancement, it is unclear which sequence is correct as neither CSF analysis, nor histopathology were performed in this patient. Slice thickness of the reformatted T1W VOL images is much thinner (0.75 mm) so may have allowed the identification of a feature that was missed on T1W SE images because of the thicker slices, (3 mm in this case). Alternatively, it may be artifactual on the T1W VOL image, due to contrast enhancement of a meningeal blood vessel. Additionally, it should be noted that the postcontrast VOL images were acquired before the postcontrast T1 SE images, and it has been shown that increased meningeal enhancement is seen immediately postcontrast compared with delayed acquisition, in normal dogs.^23^

There is a paucity of veterinary studies looking at the use of 3D GE in the clinical evaluation of canine brains. A previous study described its use for evaluation of the pituitary gland in healthy dogs.^15^ Optimal quality images were obtained with a T1W VOL sequence with a 1 mm slice thickness and 30° flip angle, before and after intravenous injection of contrast medium; these parameters are similar to those used in this study, with the exception that the study was conducted with a 0.2 Tesla open magnet.^15^

Typical data acquisition time for the T1W VOL sequence (approximately 6 min 20 s), was longer than the SE sequence (approximately 3 min 30 s). However, it is quicker than acquisition of multiple SE sequences in two planes, or the three planes that would be required to provide equivalent information. In clinical practice, it is advised that postcontrast T1W images are acquired in more than one plane.^10^ A time saving advantage can therefore be achieved using the T1W VOL sequence since the capability for subsequent orthogonal image reformatting improves acquisition efficiency.

There were several limitations to this study. Most notably, despite the adequate overall sample size (160 patients), the proportion of cases found to have lesions apparent on T1W imaging was relatively low (29). With a lower prevalence of lesions within the sample population, it is less likely that the findings would demonstrate differences between the two sequences. There was complete agreement for four of the seven parameters assessed, and the differences in lesion intensity were insufficient in number to warrant statistical analysis. Statistical analysis was performed to assess agreement for the presence or absence of meningeal enhancement with a high level of agreement demonstrated between the two sequences (*κ* = 0.906). This is however unsurprising since a difference was only identified in one case. Future studies could maximize the clinical information gained by including a greater proportion of patients with lesions, by altering the selection criteria. Secondly, assessment of the images in this study was subjective. Given the predominantly descriptive nature of the features evaluated during this observational study, and absence of objective parameters to assess, this may risk the introduction of slight observer bias.

A definitive diagnosis was not reached in many of the cases due to an absence of histopathological confirmation, and lack of relevant blood (infectious disease serology) or CSF analysis results. It is generally accepted that definitive diagnosis is not possible on the basis of MRI alone, because imaging features of neoplastic and certain nonneoplastic diseases are not sufficiently specific.^24^, ^25^ Histological examination is typically required for definitive diagnosis of intracranial neoplasms.^26^, ^27^ Magnetic resonance imaging is however regularly used for presumptive differentiation of neoplasia from inflammatory disease, and to provide probable or prioritized differential diagnoses to facilitate optimal patient management.^24^, ^25^, ^28^, ^29^, ^30^ During this study, we evaluated MRI signs that have previously been identified as being significantly associated with neoplasia, that is, a solitary lesion, presence of mass effect and contrast enhancement,^24^ extra‐axial location,^25^ and those significantly more commonly identified in inflammatory disease: meningeal enhancement and multifocal lesions.^25^ The aim of our study was however to evaluate agreement between the two sequences for assessment of several important MRI features, to determine whether a T1W VOL sequence could be used in place of the conventional sequence, rather than to evaluate accuracy for reaching a diagnosis. Additionally, the evaluation of T1W sequences alone, precludes diagnosis; concurrent assessment of other weightings, for example, T2W and FLAIR sequences, would typically be required for comprehensive evaluation of all brain lesions. The collection and inclusion of data regarding the clinical diagnosis for each patient in this study was intended to summarize the characteristics of the study population, rather than permit direct correlation with the study findings.

Based on the findings of this study, the T1W VOL technique provided comparable lesion evaluation and significantly improved GWM differentiation. The T1W VOL provided a suitable alternative T1W sequence for the evaluation of dogs with suspected intracranial disease. Therefore, T1W VOL imaging could replace conventional T1W SE for routine canine brain imaging, providing equivalent lesion appraisal and a reduction in total image acquisition time.

## LIST OF AUTHOR CONTRIBUTIONS

### Category 1

(a) Conception and Design: Fleming, Warren‐Smith
(b) Acquisition of Data: Fleming, Warren‐Smith
(c) Analysis and Interpretation of Data: Fleming, Warren‐Smith, Maddox

### Category 2

(a) Drafting the Article: Fleming, Warren‐Smith, Maddox
(b) Revising Article for Intellectual Content: Fleming, Warren‐Smith, Maddox

### Category 3

(a) Final Approval of the Completed Article: Fleming, Warren‐Smith, Maddox
